# Clinical characteristics and factors associated with triple therapy use in newly diagnosed patients with COPD

**DOI:** 10.1038/s41533-021-00227-x

**Published:** 2021-03-22

**Authors:** Mònica Monteagudo, Miriam Barrecheguren, Iryna Solntseva, Nafeesa Dhalwani, Alison Booth, Alexa Nuñez, Dimitra Lambrelli, Marc Miravitlles

**Affiliations:** 1 Primary Care University Research Institute Jordi Gol (IDIAP Jordi Gol), Barcelona, Spain; 2 Medicine Department, Universitat Autònoma de Barcelona (UAB), Barcelona, Spain; 3 Pneumology Department, Hospital Universitari Vall d´Hebron, Vall d’Hebron Institut de Recerca (VHIR), Vall d’Hebron Barcelona Hospital Campus, CIBER de Enfermedades Respiratorias (CIBERES), Barcelona, Spain; 4 Evidera, London, UK

**Keywords:** Epidemiology, Chronic obstructive pulmonary disease

## Abstract

There is limited information about the initiation of triple therapy (TT) in patients with chronic obstructive pulmonary disease (COPD) in primary care. This was an observational, population-based study in patients identified from a primary care electronic medical records database in Catalonia from 2011 to 2015 aimed to identify the use of TT in patients with newly diagnosed COPD. A total of 69,668 newly diagnosed patients were identified of whom 11,524 (16.5%) initiated TT, of whom 8626 initiated TT at or immediately after COPD diagnosis. Among them, 72.3% were GOLD A/B, 14.6% were frequent exacerbators, and 7.1% had asthma–COPD overlap (ACO). Variables associated with TT initiation were: male sex, older age, previous exacerbations, ACO, a previous treatment regimen containing an inhaled corticosteroid, previous pneumonia, and history of lung cancer. A significant number of COPD patients in Primary Care initiated TT shortly after or even before an established COPD diagnosis.

## Introduction

Treatments administered for patients with chronic obstructive pulmonary disease (COPD) in primary care do not always follow national or international guidelines^1–4^. Patients with milder forms of the disease are frequently overtreated and, although less frequently, patients with more severe COPD may be undertreated^1–4^. In a majority of countries, the initial point of contact for patients with respiratory symptoms is through primary care where targeted detection programs for the early diagnosis of COPD are supported^5,6^.

The availability of spirometry in primary care is key for early and accurate diagnosis of COPD^7^, and initial treatment should be implemented according to guidelines. The Global Strategy for Obstructive Lung Disease (GOLD) recommends classifying patients according to severity of symptoms and history of exacerbations into four categories. Initial treatment is based on one or two long-acting bronchodilators, or the combination of a long-acting beta-2 agonist (LABA) and an inhaled corticosteroid (ICS) for more symptomatic patients with frequent exacerbations^8^. When treatment with long-acting bronchodilators or with LABA/ICS is not enough to control symptoms or prevent exacerbations, treatment may escalate to triple therapy (TT) with two bronchodilators (a LABA and a long-acting antimuscarinic agent [LAMA]) and ICS. Similarly, the Spanish guidelines also recommend TT as a step up from dual bronchodilation (LABA/LAMA) or from LABA/ICS, but not as initial therapy^9^.

Studies from different countries have demonstrated a frequent use of TT in patients considered at low risk (patients with GOLD A or B disease). A recent study in the United Kingdom showed that 13.7% of GOLD A and 26.2% of GOLD B received TT^2^; similarly, data from Switzerland observed a use of TT in 13.8% of GOLD A and 28.2% of GOLD B patients^1^. It is important to understand whether this represents overtreatment of COPD or whether some of these patients could have been GOLD D patients that improved in the GOLD classification because of the efficacy of TT. In order to understand the reason for TT prescription in primary care, it is important to analyze prescription patterns in large administrative databases. The current study was conducted with the aim of characterizing newly diagnosed patients with COPD initiating TT in primary care and investigating the factors associated with TT prescription in these incident cases with COPD.

## Results

### Population of the study

**Fig. 1:**
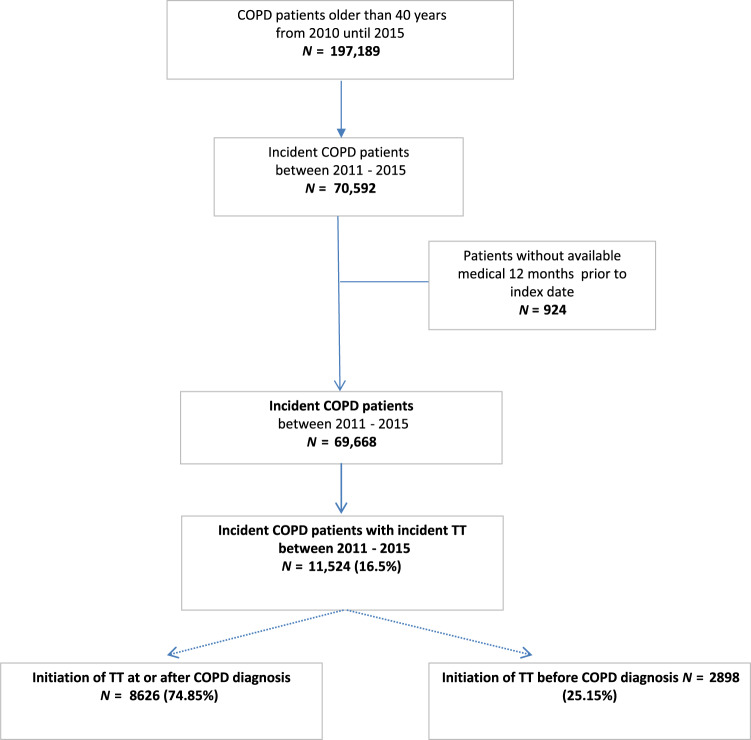
Flowchart of the study. *COPD*: chronic obstructive pulmonary disease; *TT*: triple therapy.

A total of 69,668 newly diagnosed (incident) COPD patients were identified; of whom 11,524 (16.5%) initiated TT at some point during the study period. Among these patients, 2898 (25.2%) initiated TT prior to COPD diagnosis, and 8626 (74.8%) at or after COPD diagnosis (Fig. 1).

### Clinical characteristics of patients initiating and not initiating TT, and subgroups according to TT period of initiation

The majority of incident patients with COPD were men (68.6%), with mean age of 68.3 years (standard deviation [SD]: 12.3 years). Less than half (44.3%) had spirometry tests performed within 12 months before and 2 weeks after the index date, mean FEV1 (% predicted) was 65.3% (SD: 19.6%) and 47.6% had at least one exacerbation in the previous year (Table 1).

**Table 1:** Clinical characteristics of incident COPD patients and subgroups according to TT initiation and period of initiation.

	Incident COPD patients *N* = 69,668	Incident COPD patients who do not initiate TT *N* = 58,144 (83.5%)	Incident COPD patients who initiated TT *N* = 11,524 (16.5%)	*p*	TT initiation at or after COPD diagnosis *N* = 8626 (74.8%)	TT initiation before COPD diagnosis *N* = 2898 (25.2%)	*p*
*Age, mean (SD)*	68.3 (12.3)	68.1 (12.5)	69.3 (11.3)	<0.001	68.8 (11.1)	70.8 (11.6)	<0.001
*Sex, men*	68.6	67.9	72.6	<0.001	73.6	69.4	<0.001
*Duration of follow-up in months, mean (SD)*	27.1 (16.6)	26.3 (16.5)	30.8 (16.1)	<0.001	33.8 (15.6)	21.6 (14.1)	<0.001
*Body mass index, mean (SD)*	28.7 (5.3)	28.6 (5.2)	29.3 (5.5)	<0.001	29.3 (5.5)	29.5 (5.6)	NS
*Respiratory comorbidities*							
Upper respiratory infection	24.9	25.8	20.4	<0.001	19.3	23.9	<0.001
Asthma	6.9	5.9	11.9	<0.001	9.7	18.5	<0.001
Pneumonia	6.6	6.2	9	<0.001	7.6	13.1	<0.001
Bronchiectasis	3.5	3.2	5.1	<0.001	4.3	7.7	<0.001
*Other comorbidities*							
Hypertension	53.2	52.8	55.1	<0.001	54.5	56.8	0.03
Cancer	12.5	12.3	13.6	<0.001	13	15.4	0.001
Diabetes mellitus	21.8	21.6	23.4	<0.001	22.4	26.3	<0.001
Hypercholesterolemia	44.7	45.3	42	<0.001	41.4	43.8	0.02
Anxiety	15.9	16.2	14.4	<0.001	13.9	15.8	0.01
Depression	16.1	16.2	15.4	0.05	14.7	17.6	<0.001
Osteoarthritis	22.3	22.6	20.7	<0.001	24	19.6	<0.001
*Spirometry*	44.3	46.3	34.5	<0.001	35.3	32.1	0.002
*FEV*_*1*_ *(%); mean (SD)*	65.3 (19.6)	67.1 (18.9)	55.1 (19.5)	<0.001	54.6 (19.4)	56.4 (19.6)	0.02
*Patients with exacerbations*	47.6	46.9	51.4	<0.001	49.4	57.2	<0.001
*Number of exacerbations, mean (SD)*	0.6 (0.8)	0.6 (0.82)	0.7 (0.92)	<0.001	0.7 (0.8)	0.9 (1,05)	<0.001
*Visits to primary care, mean (SD)*	8.4 (6.6)	8.1 (6.3)	9.8 (7.4)	<0.001	9.7 (7.3)	10.3 (7.7)	<0.001
*Blood eosinophil, mean (SD)*	249 (156)	248 (167)	252 (180)	NS	253 (178)	248 (188)	NS

The data are %, unless otherwise indicated. *P* values obtained by χ^2^ or *T*-Student tests.

*SD* standard deviation, *FEV*_*1*_ forced expired volume in 1 s, *LAMA* long-acting anticholinergic agent, *LABA* long-acting beta-2 agonist, *ICS* inhaled corticosteroid.

Incident patients with COPD who initiated TT more frequently had previous exacerbations (51.4 % versus 46.9%; χ^2^ test *p* < 0.001) and had more previous visits to primary care (9.8 [SD: 7.4] versus 8.1 [SD: 6.3]; *T*-test *p* < 0.001); but less frequently had available spirometry records (34.5% versus 46.3%; χ^2^ test *p* < 0.001) than patients who did not initiate TT. These differences were also observed between patients who initiated TT before diagnosis, with more exacerbations (57.2% versus 49.4%; χ^2^ test *p* < 0.001) and patients with spirometry tests (32.1% versus 35.3%; χ^2^ test *p* < 0.001) compared to patients who initiated TT at or after COPD diagnosis. Generally, patients who initiated TT prior to diagnosis also appeared to have a higher prevalence of comorbidities (particularly respiratory related) than patients who initiated TT at or after COPD diagnosis. No significant differences were observed in blood eosinophil concentration (BEC) between groups (Table 1).

### Characteristics of patients who initiated TT at or after COPD diagnosis and according to severity of disease

Among the 8626 patients who initiated TT at or after COPD diagnosis, 6237 (72.3%) were GOLD A/B and 1260 (14.6%) were frequent exacerbators. A total of 615 (7.1%) were classified as asthma–COPD overlap (ACO) (Table 2). ACO patients had a lower proportion of males (53.2%), were slightly younger 65.7 years (SD: 11.9 years), and had a higher BEC (275 cells/ml; SD: 185) than patients who did not have ACO.

**Table 2:** Characteristics of incident TT patients who initiated treatment at or after COPD diagnosis and according to severity of disease and clinical phenotypes (*n* = 8626).

	Incident COPD-TT patients *N* = 8626	GOLD A/B *N* = 6237 (72.3%)	GOLD C/D *N* = 2389 (27.7%)	ACO *N* = 615 (7.13%)	Frequent exacerbators *N* = 1260 (14.6%)	Infrequent exacerbators *N* = 3002 (34.8%)	Non-exacerbators *N* = 4364 (50.6%)
*Age, mean (SD)*	68.8 (11.1)	68.9 (11.1)	68.3 (11.3)	65.7 (11.9)	69.3 (12.2)	68.4 (11.4)	68.9 (10.6)
*Sex, men*	73.6	74.1	72.3	53.2	64	71.3	78
*Body mass index, mean (SD)*	29.3 (5.5)	29.4 (5.4)	29.02 (5.7)	29.5 (5.3)	29.8 (5.8)	29.2 (5.4)	29.1 (5.5)
*Respiratory comorbidities*							
Upper respiratory infection	19.3	16.7	26	20.3	36	20.4	13.7
Asthma	9.7	8.5	12.9	100	16.4	9.7	7.8
Pneumonia	7.6	6	11.9	7.2	18.3	10.7	2.4
Bronchiectasis	4.3	3.4	6.5	4.4	8.9	5.4	2.1
*Other comorbidities*							
Hypertension	54.5	54.3	54.8	51.9	58.1	52.2	55
Cancer	13	12.7	13.8	9.4	15.2	12.4	12.8
Diabetes mellitus	22.4	22.3	22.7	19.3	24.3	21.4	22.5
Hypercholesterolemia	41.4	40.7	43.1	38.2	46.7	39.9	40.9
Anxiety	13.9	13.5	15.2	17.7	19.2	14.1	12.3
Depression	14.7	14.5	15.3	19.8	18	15	13.6
Osteoarthritis	24	19.1	21	22.1	26.7	18.7	18.2
*Spirometry*	35.3	25.5	61.1	42.6	34.6	34.1	38.4
*FEV*_*1*_ *(%), mean (SD)*	54.6 (19.4)	66.7 (11.7)	40.7 (18.5)	59.1 (19.9)	56.1 (20.2)	54.7 (19.1)	53.8 (19.6)
*Patients with exacerbations*	49.4	41.9	69.1	59.5	100	100	0
*Number of exacerbations, mean (SD)*	0.7 (0.8)	0.42 (0.49)	1.37 (1.19)	0.88 (0.9)	2.4 (0.7)	1(0)	0
*Visits to primary care, mean (SD)*	9.7 (7.3)	9.3 (7.1)	10.6 (7.7)	9.7 (7.1)	12.7 (8.5)	9.7 (7.5)	8.8 (6.5)
*Blood eosinophil, mean (SD)*	253 (178)	254 (182)	252 (167)	275 (185)	253 (178)	254 (182)	253 (175)
*Treatment (12-month prior)*							
LAMA monotherapy	3.4	3.2	3.8	2.9)	4.1	3.5	3.1
*Combinations therapy*							
*LABA* *+* *ICS*	35.3	34.7	36.7	50.1	39.1	35.5	34.1
*LAMA* *+* *ICS*	5.7	5.5	6.2	4.7	7.3	5.9	5.4
*LABA* *+* *LAMA*	11.5	11.3	11.9	9.2	10.7	10.9	12.1
*None previous treatment*	2511 (29.1)	1864 (29.8)	647 (27.1)	162 (26.3)	343 (27.2)	875 (29.1)	1293 (29.6)

The data are %, unless otherwise indicated

*SD* standard deviation, *FEV*_*1*_ forced expired volume in 1 s, *LAMA* long-acting anticholinergic agent, *LABA* long-acting beta-2 agonist, *ICS* inhaled corticosteroid.

Regarding spirometry, it was more frequently performed in GOLD C/D patients with up to 61.1% compared to 25.5% of GOLD A/B, and these patients also had the lowest FEV_1_ (%) values (40.7%, SD: 18.5) compared to GOLD A/B (66.7%, SD: 11.7). Distribution of comorbidities was similar between subgroups (Table 2).

Patients with ACO were the group most frequently treated with a regimen including an ICS before initiating TT (54.8%) compared with the 41.2% for all the groups. About a quarter of patients had no previous treatment the year before initiating TT (Table 2).

### Time between COPD diagnosis and initiation of triple therapy

**Fig. 2:**
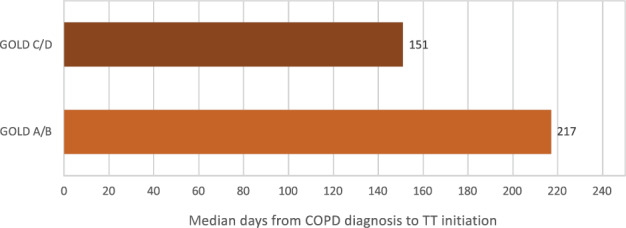
Median time from COPD diagnosis to TT according to GOLD subgroups for patients who initiate triple therapy at or after diagnosis of COPD. *COPD*: chronic obstructive pulmonary disease; *TT*: triple therapy; *GOLD*: global strategy for obstructive lung disease.

**Fig. 3:**
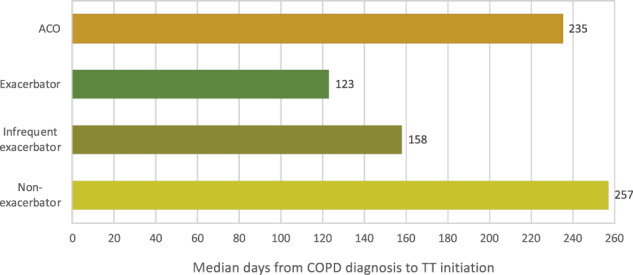
Median time from COPD diagnosis to TT according to phenotypes for patients who initiate triple therapy at or after diagnosis of COPD. *COPD*: chronic obstructive pulmonary disease; *TT*: triple therapy; *ACO*: asthma-COPD overlap.

Over the study period, median time between diagnosis and TT initiation was 49 days (interquartile range [IQR]: −1; 439 days) accounting for all patients who initiated TT at some point during the study period, before or after COPD diagnosis. Restricting the sample to patients who initiated TT at or after COPD diagnosis, median time from COPD diagnosis to TT initiation was 196 days (IQR: 15; 620 days). The median time to TT was shorter for GOLD C/D patients (151 days; IQR: 11; 535 days) compared to GOLD A/B (217 days; IQR: 18; 644 days) (Fig. 2). Regarding phenotypes, time to TT initiation was shorter in frequent exacerbators (123 days; IQR: 13; 462 days) compared to infrequent (160 days; IQR: 10; 560 days) and non-exacerbators (254 days; IQR: 122; 686 days) (Table 3 and Fig. 3).

**Table 3:** Time passed between COPD diagnosis and triple therapy treatment according to subgroups.

*Total incident TT cohort*							
Time between COPD diagnosis and TT (in days)	All (*N* = 11524)	GOLD A/B (*N* = 8224)	GOLD C/D (*N* = 3300)	ACO (*N* = 848)	Frequent Exacerbators (*N* = 2162)	Infrequent Exacerbators (*N* = 3759)	Non-exacerbators (*N* = 5603)
Median, (IQR)	49 (−1; 439)	60.5 (0; 472.7)	26.5 (−27; 349.7)	44 (−35.7; 429.5)	0 (−133.2; 196.7)	59 (0; 417)	91 (0; 539)
*Patients who initiate TT at or after diagnosis of COPD*							
Time since COPD diagnosis to TT (in days)	All (*N* = 8626)	GOLD A/B (*N* = 6237)	GOLD C/D (*N* = 2389)	ACO (*N* = 615)	Frequent Exacerbators (*N* = 1260)	Infrequent Exacerbators (*N* = 3002)	Non-exacerbators (*N* = 4364)
Median (IQR)	196 (15; 620)	217 (18; 644.5)	151 (11; 535)	235 (21; 637)	123 (13; 462)	160 (10; 560)	254 (122; 686)
*Patients who initiate TT before the diagnosis of COPD*							
Time since TT to diagnosis (in days)	All (*N* = 2898)	GOLD A/B (*N* = 1987)	GOLD C/D (*N* = 911)	ACOS (*N* = 233)	Frequent Exacerbators (*N* = 902)	Infrequent Exacerbators (*N* = 757)	Non-exacerbators (*N* = 1239)
Median (IQR)	−256 (−567.2; −82.7)	−292 (−610; −94)	−206 (−466; −71)	−303 (−674.5; −125.5)	−216 (−514; −71)	−221 (−561; −69)	−327 (−611; −108)

*IQR* interquartile range, *TT* triple therapy, *ACO* asthma–COPD overlap, *GOLD* Global initiative for Obstructive Lung Disease.

### Factors independently associated with time to TT initiation

Patients with prior treatment including an ICS showed a higher likelihood to initiate TT earlier than patients with no prior ICS treatment (Table 4). Similarly, more severe GOLD stage (C/D) (HR = 2.36, 95% CI: 2.21–2.53; logistic regression analysis *p* < 0.001), followed by ACO (HR = 1.89, 95% CI: 1.74–2.06; *p* < 0.001), and exacerbators (HR = 1.56, 95% CI: 1.52–1.61; *p* < 0.001) were associated with earlier initiation of TT. Older age, male sex, and pneumonia and lung cancer were also significantly associated with higher probability of early TT initiation (Table 4).

**Table 4:** Predictive value of the different characteristics of patients newly diagnosed with COPD for time to initiation of TT.

	Hazard ratio	95% confidence interval		*p* value
		Lower	Upper	
Age (for every year)	1.006	1.004	1.008	<0.001
Male	1.38	1.32	1.45	<0.001
Underweight <18.5 (vs normal 18.5–25)	1.40	1.11	1.78	0.004
Overweight >25–30 (vs normal 18.5–25)	1.02	0.95	1.10	0.51
Obese >30 (vs normal 18.5–25)	1.27	1.18	1.37	<0.001
Missing (vs normal 18.5–25)	1.50	1.41	1.61	<0.001
GOLD C/D (vs A/B)	2.36	2.21	2.53	<0.001
Infrequent exacerbator (vs non-exacerbator)	1.05	1.00	1.10	0.03
Exacerbator^a^ (vs non-exacerbator)	1.56	1.52	1.61	<0.001
Asthma–COPD overlap	1.89	1.74	2.06	<0.001
LAMA/ICS	2.81	2.45	3.23	<0.001
LABA/ICS	1.90	1.82	2.00	<0.001
LABA/LAMA	1.69	1.53	1.86	<0.001
Pneumonia	1.33	1.23	1.43	<0.001
Lung cancer	1.81	1.57	2.07	<0.001

All hazard ratios are adjusted for each variable presented in the table.

*LAMA* long-acting anticholinergic agent, *LABA* long-acting beta-2 agonist, *ICS* inhaled corticosteroid.

^a^Exacerbator refers to patients with at least one exacerbation the previous year.

## Discussion

The results of the current study have shown that 16.5% of patients with a new diagnosis of COPD in primary care initiated TT during the 5-year study period. Interestingly one quarter of patients initiated TT even before the diagnosis of COPD was recorded in the electronic medical records. Patients who were frequent exacerbators or had ACO were more likely to initiate TT earlier, which is in accordance with COPD treatment recommendations^8,9^. However, it appears that BEC did not influence the decision to initiate TT in this study.

In order to analyze the characteristics of patients initiating TT and time to TT initiation we focused on the patients who initiated TT at or after diagnosis of COPD. The rationale of this was to avoid any bias that may be introduced by analyzing characteristics of patients who, at the time of prescribing, might have been diagnosed with other diseases, such as asthma, or not diagnosed at all. Moreover, the group of patients initiating TT at or after diagnosis of COPD constituted three quarters of the whole population.

The observed 16.5% of incident patients with COPD initiating TT represents an increase from the 12% observed in a previous study in Spain conducted between 2007 and 2012^10^ and is higher than the 7.5% of patients initiating TT after COPD diagnosis observed in a large database study in the United States^11^. However, it is lower than the percentages observed in other European cohorts: Vetrano et al.^12^ found that 21% of patients with COPD had TT as their first inhaled prescription. In the United Kingdom, Wurst et al.^13^ observed that 23% of patients with incident COPD initiated TT, and another recent study described an increase in the use of TT as initial therapy from <1% in 2002 to 28% in 2014, with a small reduction to 22% in 2016^14^. In Italy, a 6.3% from a population of +32,000 patients with COPD in primary care were treated with TT during the first year after diagnosis^15^. It is important to contrast these figures with the current GOLD recommendations that do not include TT as initial therapy in any of the GOLD groups, but only as step up when initial therapy is not effective in controlling symptoms or preventing exacerbations^8,16,17^.

After excluding patients who initiated TT before COPD diagnosis, the remaining incident patients with COPD initiated TT a median of 196 days after diagnosis, with a shorter period observed in frequent exacerbators and GOLD C/D patients, which is in line with treatment recommendations^8^. It is of note that around one quarter of newly diagnosed COPD patients did not have any inhaled treatment before TT. Among the remaining patients, the most frequent treatment received before TT was LABA/ICS followed by LABA/LAMA. These results are different from those observed in a previous study by Monteagudo et al.^18^ in over 34,000 prevalent COPD cases with TT from the same database, in whom the most frequent previous treatment was LAMA (22.5%) followed by LABA/ICS (15.2%). Mapel et al.^19^ in the United States also found that the most frequent treatment before TT was LAMA (15.4%) followed by LABA/ICS (7.7%). It might be that incident COPD cases initiating TT may be more symptomatic and, therefore, they are more frequently started already on LABA/ICS (and early stepped up to TT) instead of starting on monotherapies. It is of note that only 34.5% of patients who initiated TT had spirometry recorded in their clinical records. The underuse of spirometry is still a pending issue in primary care that may affect diagnostic accuracy.

In multivariate analysis, we observed that some variables that define the severity of the disease were significantly associated with the initiation of TT in newly diagnosed patients with COPD. This expected finding included characteristics such as increasing age, more severe GOLD stage, and more frequent exacerbations. Another characteristic significantly associated with TT initiation was the ACO phenotype, which is also in alignment with current recommendations, that recommend the use of ICS in combination with long-acting bronchodilators to prevent exacerbations, in particular in patients with eosinophilic profile or a previous diagnosis of asthma^8,9^.

However, other identified factors associated with the initiation of TT were unexpected. Male sex was significantly associated with TT initiation in patients with incident COPD. This gender bias is difficult to explain and is not linked to the well-described diagnostic bias^20^, since all patients had a diagnosis of COPD. This finding should be analyzed in other studies that investigate whether it is due to a trend toward overprescription in men, or, alternatively an underprescription in women.

Another surprising finding is the significant association of TT initiation and the diagnosis of pneumonia. It is well recognized that the use of ICS is a risk factor for pneumonia in patients with COPD^21^ and, therefore, the opposite association would be expected. A hint to explain this paradox can be found in the next characteristic associated with initiation of TT, which is the diagnosis of lung cancer. The possible explanation could be that both patients with episodes of pneumonia and lung cancer may visit the primary care physician more frequently with more respiratory complaints, therefore, stepping up treatment to include ICS may be more likely. However, the association between the initiation of TT and pneumonia suggests that PCP may not be aware of the risk of pneumonia associated with the use of ICS, in particular in patients with more severe disease, underweight, and with low BEC^22,23^.

Finally, we did not find an association between TT initiation and BEC. Whilst it must be considered that the relevance of the use of BEC to guide ICS treatment in COPD has only appeared in recent years, the 2012 edition of the Spanish Guideline on treatment of COPD already mentioned the use of BEC to classify patients as ACO and personalize the prescription of ICS predominantly to patients with this phenotype^24^. Our data and other large database studies^18,25^ show that BEC are available for the great majority of patients with COPD in primary care and therefore can be a useful biomarker for the appropriate use of ICS in COPD^26^.

In conclusion, we have observed that around 15% of newly diagnosed patients with COPD initiate TT. Among them, one quarter had no previous inhaled therapy. Although these findings may not be consistent with current COPD guidelines, the observation that patients with ACO and frequent exacerbations were more likely to start TT and started TT earlier is in alignment with treatment recommendations.

## Methods

### Study design

This was a retrospective study with longitudinal follow-up. Data analyzed were obtained from the Information System for the Development of Research in Primary Care (SIDIAP) database; this database includes anonymized electronic medical records from 270 primary care centers with a reference population of 5.8 million people in Catalonia (Spain), which represents more than 80% of the total population^27^. This database has been used and validated for epidemiological research in respiratory diseases^25,28^.

### Patient selection

Included patients were those over 40 years of age with a first diagnosis of COPD (International Classification of Disease—10th Edition) between January 1, 2011 and December 31, 2015. Patients were required to have at least 12 months of available medical record data prior to diagnosis of COPD. Among incident patients with COPD, those who initiated TT were defined as having prescriptions for LAMA, LABA, and ICS with an overlap of 30 days or more. If a patient had a gap of 60 days for prescription of one or more of the three compounds, they were considered to no longer be persistent on TT.

### Patient subgroups

According to the time of initiation of TT, patients were classified into those who initiated TT before the date of COPD diagnosis and those who initiated TT at or after the date of diagnosis.

The phenotypes of the patients were also identified^9^. Those with a concomitant diagnosis of asthma recorded I clinical records were included in the ACO phenotype^29,30^. Patients with two or more exacerbations during the year before initiation of TT were classified as a frequent exacerbator phenotype, patients with only one exacerbation were classified as infrequent exacerbators, and the remaining COPD patients were defined as non-exacerbators. ACO and the exacerbator phenotypes were not mutually exclusive.

Patients were also classified according to the GOLD 2013 categories based on spirometry and exacerbation data as low risk (GOLD A/B) and high risk (GOLD C/D)^31^.

### Study measurements

Detailed information at the time of diagnosis was collected on the following variables: age, sex, comorbidities, smoking history, history of exacerbations, and treatments received during the year before the diagnosis of COPD. Results of spirometry and blood analysis with BEC at diagnosis were also collected.

Exacerbation episodes were identified according to previously published algorithms^10,28^. Briefly, we used a diagnostic code indicative of a respiratory exacerbation, or receipt of corticosteroids and/or antibiotics used for treating the episode. Exacerbations leading to hospitalizations or treated as part of inpatient care were not available in the data. To avoid misclassification and over-estimation of exacerbations, consecutive episodes with less than 21 days between prescriptions and physician visits were considered as a single event.

The study was approved by the Research and Ethics Committee of the IDIAP Jordi Gol Institute of Research in Primary Care (Barcelona, Spain). Since anonymized data were collected retrospectively, no informed consent was considered necessary.

### Statistical analysis

Description of variables was performed with absolute frequencies and corresponding percentages. Continuous variables were described using the mean and SD, while time from diagnosis to TT initiation in days was described using the median and IQR.

Categorical variables were compared using the χ^2^ or Fisher exact test where applicable. Quantitative variables were compared using the *T*-test or Mann–Whitney *U* test.

The variables significantly and independently associated with time to initiation of TT in newly diagnosed patients were identified by means of a logistic regression analysis. The results were described using hazard ratios with a 95% confidence interval and *p* values. All statistical analyses were performed using the statistical software package Statistical Package for Social Science (SPSS) version 20.0 (IBM, Chicago, IL, USA). A *p* value <0.05 was considered significant.

### Reporting summary

Further information on research design is available in the Nature Research Reporting Summary linked to this article.

## Supplementary information

Reporting Summary

## Data Availability

The data that support the findings of this study belong to the Catalan Healthcare System and are not publicly available. They were used under license for the current study. Data are, however, available from the corresponding authors upon reasonable request and with permission of Information System for the Development of Research in Primary Care (SIDIAP).
